# Holey-Cavity-Based Compressive Sensing for Ultrasound Imaging

**DOI:** 10.3390/s18061674

**Published:** 2018-05-23

**Authors:** Ashkan Ghanbarzadeh-Dagheyan, Chang Liu, Ali Molaei, Juan Heredia, Jose Martinez Lorenzo

**Affiliations:** 1 Mechanical Engineering Department, Northeastern University, Boston, MA 02115, USA; ghanbarzadehdaghe.a@husky.neu.edu (A.G.-D.); liu.chang4@husky.neu.edu (C.L.); j.herediajuesas@neu.edu (J.H.); 2 Electrical Engineering Department, Northeastern University, Boston, MA 02115, USA; molaei.a@husky.neu.edu

**Keywords:** compressive sensing, ultrasound imaging, ultrasound cavity

## Abstract

The use of solid cavities around electromagnetic sources has been recently reported as a mechanism to provide enhanced images at microwave frequencies. These cavities are used as measurement randomizers; and they compress the wave fields at the physical layer. As a result of this compression, the amount of information collected by the sensing array through the different excited modes inside the resonant cavity is increased when compared to that obtained by no-cavity approaches. In this work, a two-dimensional cavity, having multiple openings, is used to perform such a compression for ultrasound imaging. Moreover, compressive sensing techniques are used for sparse signal retrieval with a limited number of operating transceivers. As a proof-of-concept of this theoretical investigation, two point-like targets located in a uniform background medium are imaged in the presence and the absence of the cavity. In addition, an analysis of the sensing capacity and the shape of the point spread function is also carried out for the aforementioned cases. The cavity is designed to have the maximum sensing capacity given different materials and opening sizes. It is demonstrated that the use of a cavity, whether it is made of plastic or metal, can significantly enhance the sensing capacity and the point spread function of a focused beam. The imaging performance is also improved in terms cross-range resolution when compared to the no-cavity case.

## 1. Introduction

According to Compressive Sensing (CS) theory, unknown signals sampled at a rate smaller than that required by the Shannon–Nyquist theorem [1] may be recovered when certain conditions are satisfied. Specifically, the CS theory relies on two mathematical principles: sparsity, which imposes that the unknown signal must accept a sparse representation in a known dictionary or set of base functions; and incoherence, which requires that the Restricted Isometry Property (RIP) is satisfied by the sensing matrix that linearly relates the coefficients of the unknown signal and the undersampled data [2,3]. It can be shown that the smaller the coherence between each of two columns of the sensing matrix, the more accurately the unknown signal can be recovered [3]. When the sensing matrix is built through random projections in the undersampled signal space, the resulting coherence between each of two of its columns is small, with a high probability. This is the reason why measurement randomization has the potential to enhance the accuracy of the recovered unknown signal [2].

Several sensing and imaging applications [4] have been able to take advantage of CS by using pseudo-random illumination in the outgoing waves from the transmitters and collecting pseudo-random measurements from the incoming waves to the receivers. Carin et al. used random positions for the elements of a sensing array to make use of CS. They also showed that placing spherical scattering objects in front of the waves increases the randomness and incoherence of the measurements [5]. Incoherence between each of two measurements can also be achieved by using physical structures that exhibit different wave-matter responses at different instantaneous frequencies, without the need to change the arrangement of the sensing array. For instance, Fromenteze et al. used a metal cavity with a number of holes and a wave agitator inside the cavity to randomize the electromagnetic wave patterns for microwave imaging [6]. Later, the same research group fabricated a metalized cavity with holes arranged in irises following Fibonacci patterns to code the outgoing waves based on frequency diversity [7].

Metamaterials have also been used to create randomness in the sensing system for applications such as microwave imaging [8,9,10], optical imaging [11,12], milliliter-wave imaging [13,14,15,16] and acoustic multichannel separation using a single sensor [17]. The fabrication of holey cavities is generally simpler than that of metamaterials, and they do not require any alteration in the sensing array assortment in contrast to the approach adopted in [5]. Other methods of wave randomization have been proposed in the literature. Specifically for ultrasound imaging, Schiffner introduced a software-based technique that uses time delays and apodization weights to generate random incident acoustic fields [18,19]. Other software methods, which usually involve novel sampling methods, have been proposed, as well [20,21]. Similar to coded masks that are commonly used in compressive optical imaging [22,23,24] as a subgroup of hardware-based methods, Kruizinga et al. introduced a rotating mask of randomly-varying thicknesses throughout its surface to randomize ultrasound waves, and they were able to retrieve 3D images of objects using a single transducer [25]. In this study, a static 2D cavity has been selected as a structure that enables randomization of the wave fields in three generalized dimensions, one spectral and two spatial, thus leading to enhanced ultrasound imaging via compressive sensing. Other types of ultrasound cavities have also been proposed by Fink et al. to create images using time-reversal techniques [26,27,28,29]. However, they have not been used in the scope of compressive sensing; and therefore, their method requires a large number of measurements [25]. In the succeeding sections, the performance of several 2D holey cavities are studied, showing how the cavity-based imaging performance is enhanced when compared to that of a traditional ultrasound imaging setup.

## 2. Two-Dimensional Cavity

**Figure 1:**
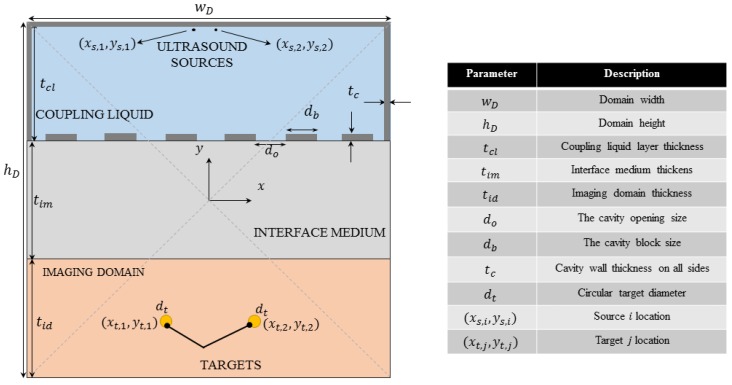
The simulation domain on the left and the geometrical parameters on the right. From the list of parameters, the size of the openings at the bottom of the cavity and the material used to fabricate the cavity are subject to change.

In order to design the cavity, a baseline configuration is defined as shown in Figure 1. It includes a coupling liquid region that contains the ultrasound sources, an interface region and an imaging region that contains the unknown targets surrounded by a known homogeneous background. In many applications, the ultrasound probe is not in direct contact with the imaging domain; therefore, the interface medium was introduced to take into consideration this fact. One example of a 2D cavity enclosing the exciting sources is shown in the top layer of Figure 1. As observed, the cavity is closed from all sides except the bottom, where a number of openings is made for the impinging waves to pass through. It is assumed that the openings are uniformly distributed along the bottom of the cavity, and they are symmetric with respect to the *y* axis.

The parameters listed on the right side of Figure 1 are adjustable. In the next section, the theory required to solve the acoustic problem is presented; and later, in Section 4, a simulation is carried out for a selected set of geometrical and physical parameters using the established theory.

## 3. Compressive Sensing, Imaging and Performance Metrics

In this section, the algorithms used to perform compressive sensing and imaging are introduced. Specifically, a computational forward model is used to simulate the sensing process and measured acoustic wave fields, the inverse model is formulated and the first-order Born approximation is used as a simple approach to linearize the imaging problem. Moreover, the sensing capacity and the Point Spread Function (PSF) of the system are defined, and they will be used as extra metrics to assess how the addition of the cavity affects the performance of the imaging system.

### 3.1. The Forward Model

For an inhomogeneous medium, with its density and speed of sound defined as a function of the location vector r, the linear wave equation for a time-harmonic pressure field can be expressed as follows [30]:

(1) $$\rho \left(r\right)\nabla \;\cdot \left(\frac{1}{\rho (r)}\nabla \;P\left(r,\omega \right)\right)+k^{2}\left(r,\omega \right)P\left(r,\omega \right)=F\left(r,\omega \right),$$

in which ω=2πf is the angular frequency with *f* denoting the frequency, ρ(r) is the density, k(r,ω) is the wavenumber, P(r,ω) is the pressure field and F(r,ω) is the acoustic source term, all defined in the medium. The mathematical model of F(r,ω) depends on how the source is defined. If the exciting source is a monopole, and it is defined based on the Root Mean Square (RMS) of power per unit length, i.e., $P_{rms}$, then [31]

(2) $$F\left(r,\omega \right)=2e^{i\phi_{s}}\sqrt{2\rho \left(r\right)\omega \;P_{rms}}\delta \;\left(r-r_{s}\right)$$

where $\phi_{s}$ and $r_{s}$ are the phase and the location of the source, respectively, and $\delta \;(r-r_{s})$ is the Dirac delta function, shifted from the origin to the source location. To excite a delta function having a magnitude of one (pascals per unit area) and no phase terms, the parameters must be chosen as $\phi_{s}=0$ and $P_{rms}\left(\omega \right)=1/\left(8\rho \left(r\right)\omega \right)$. Equation (1) is solved for the complete geometry shown in Figure 1 using the finite-element software COMSOL Multiphysics (CM), one time with the targets present and one time when they are absent. The former case gives the background field, and the later one gives the total field, both of which will be used in the inverse model to retrieve the image of the targets, as will be discussed in the succeeding subsection. The outer boundaries of the entire simulation domain on all sides are set to be absorbing. Moreover, since the Pressure Acoustics Module of CM is used, the cavity and other solids in the medium are viewed as fluids by the module in terms of wave propagation. In this way, each material in the simulation domain is characterized by its density and longitudinal speed of sound. This method offers an approximation of solid behavior without the need to use the Solid Mechanics Module, and it has been previously used in the literature [32].

### 3.2. The Inverse Model

The computed total and background fields in the forward modeling inside the imaging domain can be denoted with ${\tilde{P}}_{b}$ and ${\tilde{P}}_{t}$. When the targets are small and the density gradient ∇ρ(r) between the targets and the homogeneous background medium is relatively small, ${\tilde{P}}_{b}$ and ${\tilde{P}}_{t}$ both satisfy the homogeneous and approximated version of Equation (1):

(3) $$\left(\nabla^{2}+k_{b}^{2}\left(r,\omega \right)\right)\tilde{P_{b}}\left(r,\omega \right)=0,$$

(4) $$\left(\nabla^{2}+k_{t}^{2}\left(r,\omega \right)\right)\tilde{P_{t}}\left(r,\omega \right)=0,$$

which are in the form of the linear, homogeneous Helmholtz equations. Here, $k_{b}\left(r,\omega \right)$ and $k_{t}\left(r,\omega \right)$ denote the wavenumbers in the absence and presence of the targets, respectively. If the scattered field is defined as $\tilde{P_{s}}\left(r,\omega \right)=\tilde{P_{t}}\left(r,\omega \right)-\tilde{P_{b}}\left(r,\omega \right)$, then Equation (3) can be rewritten as:

(5) $$\nabla^{2}\left(\tilde{P_{t}}\left(r,\omega \right)-\tilde{P_{s}}\left(r,\omega \right)\right)+k_{b}^{2}\left(r,\omega \right)\left(\tilde{P_{t}}\left(r,\omega \right)-\tilde{P_{s}}\left(r,\omega \right)\right)=0.$$

**Figure 2:**
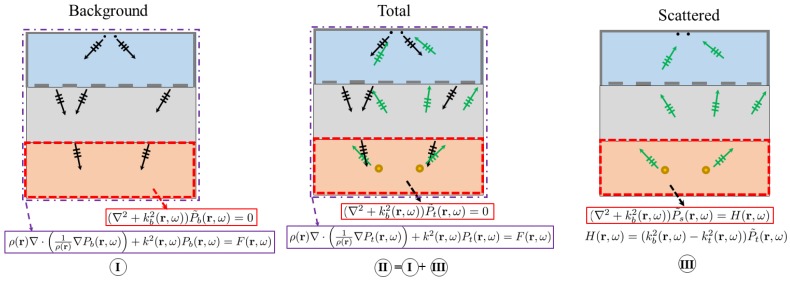
The background, total and scattered pressure fields for a monopole acoustic source all satisfy the Helmholtz equation in the imaging domain, provided that the density of the scattering target is close to that of the background.

Next, subtracting Equation (5) from Equation (4), one can easily show that:

(6) $$\left(\nabla^{2}+k_{b}^{2}\left(r,\omega \right)\right)\tilde{P_{s}}\left(r,\omega \right)=\left(k_{b}^{2}\left(r,\omega \right)-k_{t}^{2}\left(r,\omega \right)\right)\tilde{P_{t}}\left(r,\omega \right),$$

which is similar to (3) and (4), except the exciting source $H\left(r,\omega \right)=\left(k_{b}^{2}\left(r,\omega \right)-k_{t}^{2}\left(r,\omega \right)\right)\tilde{P_{t}}\left(r,\omega \right)$ and the subscript *s*, which stands for the scattered field. The application of the Helmholtz equation in the background, total and scattered field is summarized in Figure 2. The solution to Equation (6) can be obtained by superposition as [33]:

(7) $$\tilde{P_{s}}\left(r,\omega \right)=\int_{S_{id}}G_{b}\left(r,r^{\prime },\omega \right)H\left(r^{\prime },\omega \right)dr^{\prime }=\int_{S_{id}}G_{b}\left(r,r^{\prime },\omega \right)k_{b}^{2}\left(r^{\prime },\omega \right)X\left(r^{\prime },\omega \right)\tilde{P_{t}}\left(r^{\prime },\omega \right)dr^{\prime },$$

in which $G_{b}\left(r,r^{\prime },\omega \right)$ is the solution of the background pressure when the exciting source is an impulse function $-\delta (r-r^{\prime })$ at location $r^{\prime }$; $X\left(r,\omega \right)=\left(1/c_{b}^{2}\left(r,\omega \right)-1/c_{t}^{2}\left(r,\omega \right)\right)/\left(1/c_{b}^{2}\left(r,\omega \right)\right)$ is the contrast variable and the relationships $k_{b}\left(r,\omega \right)=\omega /c_{b}\left(r,\omega \right)$ and $k_{t}\left(r,\omega \right)=\omega /c_{t}\left(r,\omega \right)$ are used with $c_{b}\left(r,\omega \right)$ and $c_{t}\left(r,\omega \right)$ being the speed of sound inside the domain in the presence and the absence of targets, respectively. The subscript $S_{id}$ denotes the area on which the integrand is integrated, which is the imaging domain. It should be noted that inside the imaging domain, X(r,ω) is zero everywhere, save the inside and on the boundaries of the targets. Green’s function $G_{b}\left(r,r^{\prime },\omega \right)$ is also calculated numerically using a full-field simulation for each frequency and for the entire simulation domain, including the cavity, but without the targets.

The introduced parameter $X(r^{\prime },\omega )$ is to be solved for in the imaging problem as the unknown. Provided that the speed of sound does not vary much in the frequency band used [34], the dependence of the contrast variable on frequency may be neglected, i.e., $X\left(r^{\prime },\omega \right)\approx X\left(r^{\prime }\right)$. When the contrast variable is relatively small, causing a small perturbation in the fields, the total field can be estimated from the background field using the first-order Born approximation [35,36], which turns Equation (7) into:

(8) $$\tilde{P_{s}}\left(r,\omega \right)\approx \int_{S_{id}}G_{b}\left(r,r^{\prime },\omega \right)k_{b}^{2}\left(r^{\prime },\omega \right)X\left(r^{\prime }\right)\tilde{P_{b}}\left(r^{\prime },\omega \right)dr^{\prime },$$

where $P_{b}\left(r^{\prime },\omega \right)$ is to be numerically computed and $\tilde{P_{s}}\left(r^{\prime },\omega \right)$ is obtained by subtracting the simulated total field and background field at the position of the receivers.

### 3.3. Distributed Compressive Sensing and Imaging Algorithm

Discretizing Equation (8) into *P* pixels in the imaging domain and having *M* noiseless measurements on the receivers leads to the following matrix-form equation:

(9) $$A_{\left(M\times P\right)}{\hat{x}}_{\left(P\times 1\right)}=b_{\left(M\times 1\right)},$$

in which b is the measurement vector, A is the sensing matrix and $\hat{x}$ is the estimated unknown vector—the column-wise stacking of the values of the contrast parameter at each pixel in the imaging domain that will be later reshaped into the actual size of the that domain to give the 2D image. The number of pixels *P* is determined by the mesh grid in which the fields are exported; and it equals $n_{x}n_{y}$, where $n_{x}$ and $n_{y}$ are the size of the grid in the *x* and *y* direction, respectively (Figure 1). If the number of transmitters, receivers and the frequencies used in the measurements are respectively denoted by $N_{T}$, $N_{R}$ and $N_{f}$, the number of measurements equals $M=N_{T}N_{R}N_{f}$, which is explained in details in the Appendix. The system of equations in (9) is underdetermined, since the number of measurements *M* is usually much smaller than the number of unknowns *P*. Therefore, an optimization method is needed to find an optimal solution for the system out of infinitely many solutions, particularly in this case where compressed sensing is considered. A norm-one optimization technique called the Alternating Direction Method of Multipliers (ADMM) is employed to find an optimized solution for $\hat{x}$ in a fully-distributed fashion. The version of the ADMM used in this study makes use of parallel computing [37] in *Q* levels, and it is formulated as follows [38,39]:

(10) $$\left.\begin{array}{c} \mathrm{minimize}\;\;\;\sum_{i=1}^{Q}{∥A_{i}{\hat{x}}_{i}-b_{i}∥}_{2}^{2}+\eta \;{∥z∥}_{1}\\ \;\;\;\mathrm{subject}\;\;\;\mathrm{to}\;\;\;\;{\hat{x}}_{i}-z=0\;\;\;\;\;\forall i=1,\ldots ..,Q; \end{array}\right.$$

in which matrix A has been segmented based on rows into *Q* submatrices $A_{i}$, ${\hat{x}}_{i}$ is the solution provided by the submatrix $A_{i}$, $b_{i}$ is the subvector of b that is associated with $A_{i}$, η is the norm-one regularization parameter and z is the consensus solution obtained after combining all solutions ${\hat{x}}_{i}$. The Lagrangian form of Equation (10) is as follows [37] (§3.1):

(11) $$\begin{array}{cc} L_{\rho }\left({\hat{x}}_{1},\cdots ,{\hat{x}}_{Q},\hat{z},{\hat{u}}_{1},\cdots ,{\hat{u}}_{Q}\right) & =\frac{1}{2}\sum_{i=1}^{Q}{∥A_{i}{\hat{x}}_{i}-b_{i}∥}_{2}^{2}+\eta {∥\hat{z}∥}_{1}\\ & +\frac{\rho }{2}\sum_{i=1}^{Q}{∥{\hat{x}}_{i}-\hat{z}+{\hat{u}}_{i}∥}_{2}^{2}-\frac{\rho }{2}\sum_{i=1}^{Q}{∥{\hat{u}}_{i}∥}_{2}^{2}, \end{array}$$

where ρ is the augmented Lagrangian parameter and ${\hat{u}}_{i}$ is the scaled form of the Lagrangian multiplier [37] (§3.1.1). This problem can be optimized iteratively as presented in Algorithm 1.
**Algorithm 1** Consensus ADMM. **Inputs:** A← sensing matrix b← measurement vector $n_{max}$← maximum number of iterations Q← number of rows divisions ρ← augmented Lagrangian parameter η← norm-one regularization parameter Initialize ${\hat{x}}_{1}^{\left(0\right)}={\hat{x}}_{2}^{\left(0\right)}=\cdots ={\hat{x}}_{Q}^{\left(0\right)}={\hat{z}}^{\left(0\right)}={\hat{u}}_{1}^{\left(0\right)}={\hat{u}}_{2}^{\left(0\right)}=\cdots {\hat{u}}_{Q}^{\left(0\right)}=0$▹ Initialization Compute the inverse factor $\Psi_{i}={\left(A_{i}^{*}A_{i}+\rho I\right)}^{-1}$ for i=1,⋯,Q k←0 iteration number **repeat**   ${\hat{x}}_{i}^{\left(k+1\right)}=\Psi_{i}\times \left(A_{i}^{*}b_{i}+\rho \left({\hat{z}}^{\left(k\right)}-{\hat{u}}_{i}^{\left(k\right)}\right)\right)$ for i=1,⋯,Q▹ Update ${\hat{x}}_{i}^{\left(k+1\right)}$   ${\bar{x}}^{\left(k+1\right)}=\frac{1}{N}\sum_{i=1}^{Q}{\hat{x}}_{i}^{\left(k+1\right)}$▹ Mean of ${\hat{x}}_{i}^{\left(k+1\right)}$   ${\bar{u}}^{\left(k\right)}=\frac{1}{N}\sum_{i=1}^{Q}{\hat{x}}_{i}^{\left(k\right)}$▹ Mean of ${\hat{x}}_{i}^{\left(k\right)}$   ${\hat{z}}^{\left(k+1\right)}=S_{\eta /\rho Q}\left({\bar{x}}^{\left(k+1\right)}+{\bar{u}}^{\left(k+1\right)}\right)$▹ Update $z^{\left(k+1\right)}$   ${\hat{u}}_{i}^{\left(k+1\right)}={\hat{u}}_{i}^{\left(k\right)}+\left({\hat{x}}_{i}^{\left(k+1\right)}-{\hat{z}}^{\left(k+1\right)}\right)$ for i=1,⋯,Q▹ Update $u_{i}^{\left(k+1\right)}$   k←k+1▹ Increment *k* **until**
$k>n_{max}$▹ Check for convergence **Output:**
$z^{\left(k+1\right)}$

In the algorithm, I denotes the identity matrix, $\bar{x}$ and $\bar{u}$ are, respectively, the mean values of ${\hat{x}}_{i}$ and ${\hat{u}}_{i}$ for all *i* and $S_{\frac{\eta }{\rho Q}}\left(\cdot \right)$ is the soft thresholding operator, which is applied on each element of the vector. When it applies on a scalar β, it functions as [39,40]:

(12) $$S_{\frac{\eta }{\rho Q}}\left(\beta \right)=\left\{\begin{array}{cc} \beta -\frac{\eta }{\rho Q}\mathrm{sign}\left(\beta \right) & |\beta |>\frac{\eta }{\rho Q},\\ 0 & \left|\beta \right|\leq \frac{\eta }{\rho Q}. \end{array}\right.$$

To calculate the inversion of $\Psi_{i}=A_{i}^{*}A_{i}+\rho I$, where $A_{i}^{*}$ denotes the Hermitian of the sensing matrix, more efficiently, the matrix inversion lemma can be used, as described in [38].

When there is noise in the measurements, Equation (9) can be rewritten as:

(13) $$A{\hat{x}}_{n}=\tilde{b}=b+\tilde{n},$$

where $\tilde{n}$ is the noise column vector, having the same size as b, $\tilde{b}$ is the measurement vector including noise and ${\hat{x}}_{n}$ is signal retrieved from noisy measurements. The imaging results using both noiseless measurements and measurement including noise will be presented in the Section 4.

### 3.4. Beam Focusing

The performance of the sensing array with and without the cavity can be seen through its PSF, which is the field distribution that results from coherently adding up the pressure field of each transmitter (or receiver), multiplied by a phase term that produces a constructive interference at the focusing point, when the transmitter (or receiver) is excited by a unit impulse function. The described procedure is named phase-based beam focusing, and the resulting PSF for transmitters is given by the following equation [13,41]:

(14) $$BF_{p}\left(r\right)=\sum_{i=1}^{N_{T}}\sum_{l=1}^{N_{f}}P_{b,il}\left(r,f_{l}\right)e^{-j\psi_{p,il}},$$

in which $P_{b,il}\left(r,f_{l}\right)$ is the background field due to transmitter *i* at location r at frequency $f_{l}$ and $\psi_{p,il}$ is the phase of the background field at the location of the focus point $r_{p}=\left(x_{p},y_{p}\right)$, i.e., $∡P_{b,il}\left(r_{p},f_{l}\right)$.

### 3.5. Sensing Capacity

Another metric that will be used to assess the performance of the imaging system is the so-called sensing capacity. This metric determines the amount of information that can be transferred from the imaging domain into the sensors; and the larger the sensing capacity is, the better the image reconstruction will be. The sensing capacity, expressed in bits/s/Hz, is computed as follows [41,42]:

(15) $$C=\Sigma_{i=1}^{q}\log_{2}\left(1+r\zeta_{i}^{2}\right),$$

where *r* is the Signal-to-Noise Ratio (SNR), $\zeta_{i}$ is the *i*-th nonzero singular value of the sensing matrix—when the values are arranged in ascending order, and *q* is the number of active transceivers. As observed, *C* is closely related to the singular values of the sensing matrix, which itself is contingent upon the sensing array and how it has been configured.

## 4. Simulation Results and Discussion

The goal of this section is to compare the performance of the imaging system with and without the cavity. Specifically, the performance will be assessed in terms of the sensing capacity, the PSF of a focused beam and the image quality. A typical ultrasound imaging medium, which satisfies both the density and contrast conditions, is used as a case study in this paper according to the geometry shown in Figure 1; see Table 1 and the references therein for material properties. As shown in Figure 1, a small number of transceivers (only two) are considered here to show the general concept and the imaging capability using compressive sensing. What is more, two point-like targets are selected to be imaged. Based on the values in Table 1, one can observe that: (i) the contrast variable takes a relatively small value (about 7.84%) at the location of the targets, and it is equal to zero in the background medium; (ii) the targets are comparatively small compared; and (iii) the density difference between the targets and the background medium is about 4%. Thus, the approximations made to obtain Equation (8) can be assumed to be valid and it will be shown that this is indeed the case in the imaging results.

To export the fields, the simulation domain is discretized into a mesh grid size of 501×501. The imaging domain has a grid size of $n_{x}=501$ and $n_{y}=120$ in the *x* and *y* direction, in order, leading to a vector signal size of P=60120 elements. The frequency band is 2–10 MHz, and the frequency steps in the sweeping are 0.1 MHz. The number of transmitters $N_{T}$, receivers $N_{R}$, and frequencies $N_{f}$ used in the simulations is 2, 2 and 81, which yields a total measurement number of $M=N_{T}N_{R}N_{f}=324$. It is lucid that these values for *M* and *P* make the system in (9) underdetermined since P≫M.

### 4.1. The Effect of the Cavity Design on Sensing Capacity

As introduced in Section 2, there are a number of parameters that can be adjusted in the cavity design. Thus, before any imaging or beam focusing is performed, the cavity parameters are required to be selected. In this study, maximizing the sensing capacity was selected as the design goal, and two parameters of the cavity were adjusted to achieve this end: the opening size and the material of the cavity. The numeric values of the geometric parameters used in the simulations, except $d_{o}$ and $d_{b}$, which are variable in the cavity design, are given in Table 2.

#### 4.1.1. The Size of the Openings

To have the ability to sample all the cavity modes, the size of the openings in the cavity needs to be smaller than the minimum guided wavelength [6]. The frequency band for the simulations in this study is selected to be 2–10 MHz, which covers the typical frequencies used in high resolution ultrasound devices [52]. Hence, the opening size is limited by the lowest wavelength in water corresponding to the highest frequency, that is $\lambda_{min}=c_{water}/f_{max}=1490/1\times 10^{6}=0.149$ mm, with $c_{water}$ being in m/s and $f_{max}$ in Hz. On the other hand, the hardships in fabrication and micromachining set a limit on how small the openings can be.

**Figure 3:**
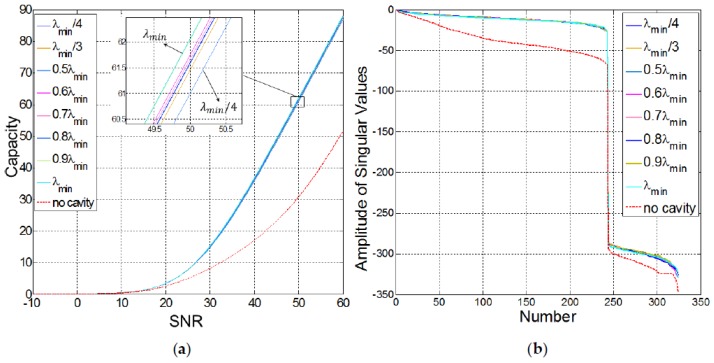
(**a**) Sensing capacity in bits/s/Hz and (**b**) the amplitude of the singular values of the sensing matrix in dB for different opening sizes, in terms of $\lambda_{min}$, in the cavity against the case where no cavity is used. SNR is also represented in dB.

Eight different cases are studied, in which the cavity thickness and its material (steel) are kept constant as the size of the holes at the bottom of the cavity was changed. The number of holes is maximized in each case by fitting as many openings as possible at the bottom of the cavity, with the assumption that $d_{o}=d_{b}$. Moreover, the openings are generated symmetrically with respect to the *y* axis, as mentioned before. The results of the sensing capacity versus SNR are shown in Figure 3. It is evident that the addition of the cavity to the simulation domain has increased the sensing capacity; however, the alterations in the opening sizes have not made a significant difference, at least in the dB units used in the plots. With this setup, the largest opening size ($\lambda_{min}$) has resulted in the largest sensing capacity among other opening sizes, and at the same time, it is the easiest to fabricate in terms of feature size.

#### 4.1.2. Material Selection

**Figure 4:**
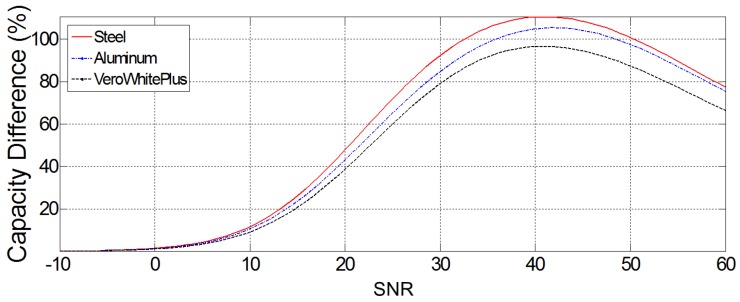
Comparing the sensing capacity difference (%) of the steel, aluminum and VeroWhitePlus cavities with the no-cavity case in the setup shown in Figure 1. SNR is in dB.

3D printing technology has made prototype fabrication much easier for many applications [53,54,55]. Hence, it is of interest to inspect whether using a 3D printing material such as VeroWhitePlus, which is not as stiff and dense as steel, can adequately randomize the wave fields for compressive sensing. Furthermore, another material, aluminum, is tested as the cavity material, and its effect on the sensing capacity is studied, alongside with that of steel. The results, in terms of sensing capacity difference with the no-cavity case, are illustrated in Figure 4. The sensing capacity difference is defined as below:

(16) $$\frac{C_{cavity}-C_{no\;cavity}}{C_{no\;cavity}}\times 100$$

in which $C_{cavity}$ and $C_{no\;cavity}$ are the sensing capacity values obtained by (15) in the presence and absence of the cavity, respectively.

**Figure 5:**
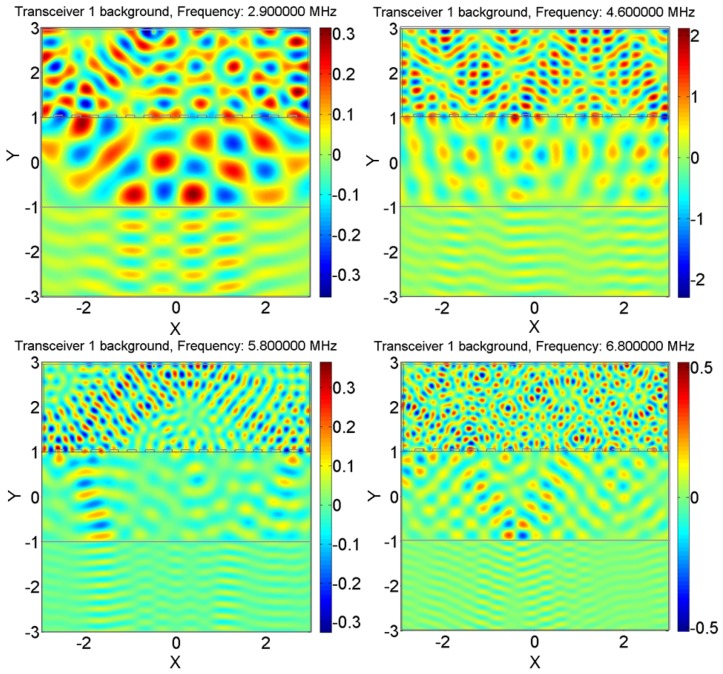
Wave pattern coding using the aluminum cavity around the transceivers. Different and psuedo-random modes are excited at each frequency due to the presence of the holey cavity and the interaction of the waves with it. The pressure units are in pascals, and the amplitude of the source excitation was set to be 1 Pa. X and Y are in mm.

Although the addition of the plastic cavity to the domain has increased the sensing capacity, its effect is not as strong as that of the steel or the aluminum cavity. Since aluminum is lighter than steel, it can be made into thin layers, and its effect in the cavity is close to that of steel, so it was selected as the material for the cavity. It is noteworthy to remark that the maximum capacity difference occurs when the SNR is about 40 dB. Figure 5 shows the wave pattern randomization at four different frequencies in the 2–10-MHz interval for the aluminum-cavity case. It is easy to observe that the field patterns have a reduced correlation as a result of pseudo-random illumination of the scene.

### 4.2. The Effect of the Cavity on Imaging and Point Spread Function

**Figure 6:**
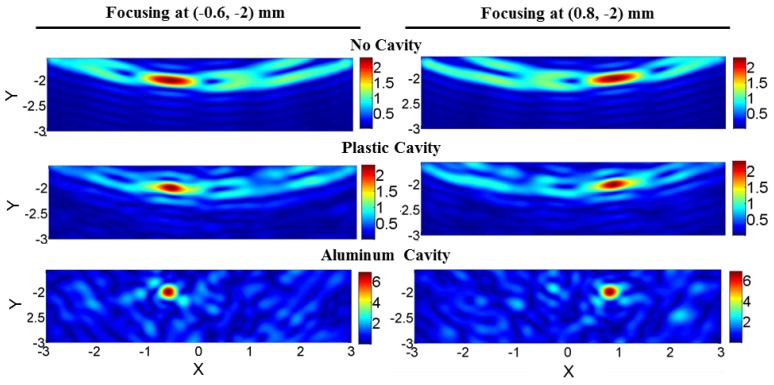
The PSF when the phase-based focused beam is formed at (−0.6,−2) mm and (0.8,−2) mm on the left and right side, respectively: without a cavity, with the plastic cavity and with the aluminum cavity (from top to bottom). Focusing is strongly improved in the case of the aluminum cavity. The unit of focused beams is in pascals, and *X* and *Y* are in mm. It should be noted, as described in Section 3.4, that these PSFs are formed using the background fields without any targets present.

The cavity effect on the PSF of a focused beam is shown for three cases in Figure 6: (i) without the cavity, (ii) when a plastic cavity encloses the sources and (iii) when an aluminum cavity encloses the sources. The PSF of the imaging system improves considerably when the aluminum cavity was used. Specifically, the cross-range aliasing effects are eliminated, and the cross-range resolution is enhanced; nevertheless, this enhancement is not as significant when the plastic cavity is employed.

**Figure 7:**
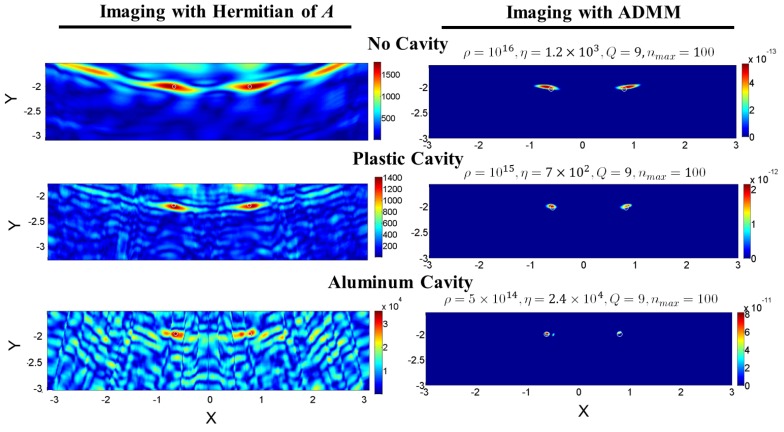
Imaging of the point-like targets with the Hermitian of the sensing matrix and with the ADMM: without the cavity, with the plastic cavity and with the aluminum cavity. Measurement randomization has enhanced the cross-range resolutions in both cases of the plastic and aluminum cavity, but more effectively in the later case than the former. The addition of the aluminum cavity has introduced a weak artifact beside the left target; yet, the location of both targets is clear, showing the maximum intensity. These graphs illustrate the retrieval results of the contrast variable, which is unitless. *X* and *Y* are in mm. The ADMM input parameters (Algorithm 1) are also presented at the top of each ADMM image.

Figure 7 illustrates the images created from noiseless measurements for the same three cases, alongside the ADMM input parameter values for each case. The maximum number of iterations $n_{max}$ is selected such that the convergence is ensured, and the number of row divisions *Q* is selected such that a relatively quick consensus-based solution is achieved. Selecting *Q* to be relatively small increases the computational speed in the variable convergence while increasing the computation time in the initial matrix inversions; yet, selecting it to be relatively large could unnecessarily elevate the computational load and time in overall, as many parameters must reach consensus.

**Figure 8:**
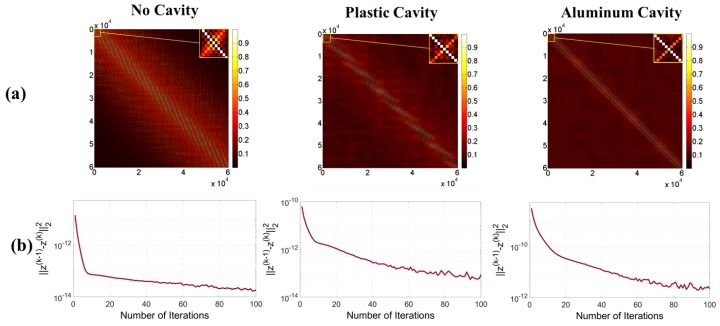
(**a**) The multiplication of the sensing matrix by its Hermitian, normalized to have a maximum value of one (as the criterion for the quality of approximation), and (**b**) the convergence plot of the ADMM images presented in semi-log format for a better view of the values. The convergence plots show that the difference between the absolute value of $z^{\left(k-1\right)}$ and $z^{\left(k\right)}$ (refer to Algorithm 1) quickly decreases to relatively minute values, which ensures reaching the convergent solution. The convergence of the solution of the cavities case shows some noticeable oscillations, which are permissible, as they are small relative to the final error value.

The selection of ρ generally depends on factors such as the numerical values of the elements of A, and η is chosen to sparsify the obtained image. These values are chosen with the prior knowledge of the ground truth and using a trial-and-error approach, while the most sparse, converging solution is sought. Looking at the first step of the iteration in Algorithm 1 shows that for ρ to have an effect on the value of $\Psi_{i}$, its value should be comparable to the absolute value of $A_{i}^{*}A_{i}$ elements on the main diagonal. Thus, the absolute value of the largest element of $A_{i}^{*}A_{i}$, which lies on the main diagonal (Figure 8), can be used as a first guess for the order of ρ. Then, several values for ρ, multiple orders of magnitude below or above the absolute value of the largest element of $A_{i}^{*}A_{i}$, are tested, while η is set to zero. Next, the value of η is tuned, and later fine-tuned, so that the most sparse solution is achieved, while making sure the peaks of the sought signal, which come from the targets, are kept.

**Figure 9:**
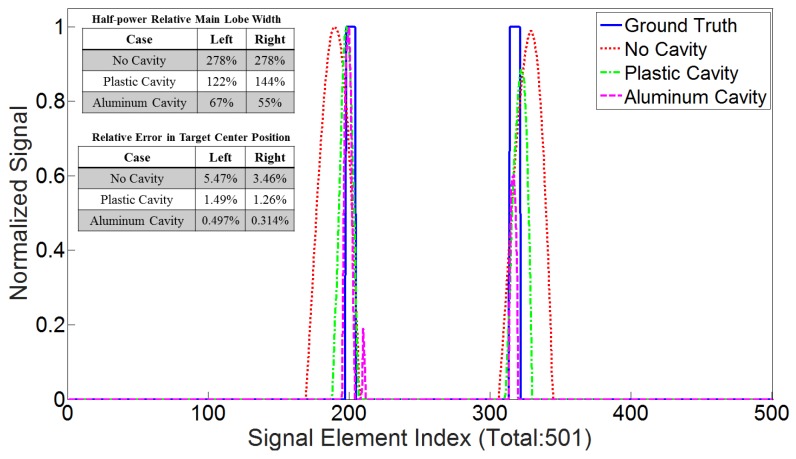
Inspection of the retrieved signals at y=−1.98 mm and their comparison with the known ground truth. The relative half-power width of the signals’ main lobes on the left and right are computed with respect to the width of the main lobes of the ground truth signal. The outcomes are presented at the top-left of the plot, showing that the aluminum cavity, despite having side lobes, leads to the best cross-range resolution among the others. Using this cavity has also outperformed other methods in terms of the relative error generated in the target center position.

To better show how well the imaging in each case has performed, the normalized signals restored by the ADMM at the line passing through y=−1.98 mm are plotted separately in Figure 9, alongside the known ground truth. The normalization is done with respect to the maximum value of each signal, and the horizontal axis shows the signal index or the pixel number in the *x* direction. The Relative Half-Power Width (RHPW) (equivalent to a −3-dB width when the signals are presented in decibels) of each main lobe of all the signals is also computed with respect to the width of the main lobes in the ground truth. The relative error in the prediction of the center of the targets for the no-cavity, plastic-cavity and aluminum-cavity case respectively were 5.47%, 1.49% and 0.497% for the left target and 3.46%, 1.26% and 0.314% for the right target. The RHPW of the signal retrieved when the aluminum cavity was used is the narrowest among the signals reconstructed from other methods. In this case, the fact that the main lobe widths have fallen below the ground truth is due to the high value of η that was required to make the solution as sparse as possible, without losing the signal from each target.

The results generated by the ADMM are also compared to those provided by traditional Hermitian matrix imaging in Figure 7. The Hermitian imaging is based on approximating the solution to Equation (9) by $\hat{x}\approx A^{*}b$, where $A^{*}$ is the complex-conjugate transpose or Hermitian of matrix A. In this imaging technique, it is assumed that $A^{*}A\approx \gamma \;I$, where I is the identity matrix and γ is a constant. The extent to which this assumption is valid needs to be determined by evaluating how close the magnitude of $A^{*}A$, when normalized, is to the identity matrix, which will be shown below. Since the results of this approximation provide an additional insight into the imaging performance of the system, they are presented besides the images retrieved by the ADMM.

Figure 8 shows that the matrix $A^{*}A$, when normalized, is closest to I when the aluminum cavity used, thus leading to the best imaging of all configurations. It is also important to note that, for the ADMM imaging, the residual error in the first iteration for the no-cavity, plastic cavity and aluminum cavity case is $1.35\times 10^{-11}$, $6.312\times 10^{-11}$ and $2.98\times 10^{-9}$, respectively. In spite of these challenging initial conditions, the ADMM algorithm can retrieve the signal and it shows that the aluminum cavity case leads to the best imaging performance after 100 iterations. The main reason why the ADMM method produces solutions that are 20 orders of magnitude smaller than those of the Hermitian method is because the former inverts the linear system of equations described in Equation (10), while the latter just multiplies both sides of the equation by the transposed conjugate matrix—in other words, the linear system of equations is not inverted. This can also be seen from the fact that in the Hermitian method, $A^{*}A$ is not approximately equal to the identity matrix I, but a scaled version of it, γI. The constant γ makes the solution of both methods different. In addition to that, the regularization parameters impose a solution that is sparse; and depending on the sparsity level, the amplitude of the reconstructed contrast function changes. In any case, the normalized images from both methods would be a suitable reconstruction of the size and location of the targets.

**Figure 10:**
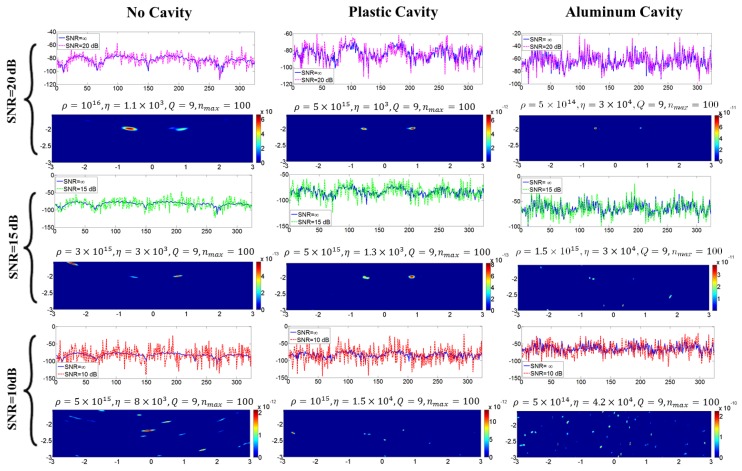
The study of adding noise to the measurements: rows showing the ADMM imaging results with different SNRs of 20 dB, 15 dB and 10 dB and columns showing different case studies. With this configuration, the plastic cavity is the most adaptive to noise.

The imaging results discussed so far did not account for any noise in the measurements. Figure 10 shows the results when noise is included for different signal-to-noise ratios. The noise was generated with MATLAB’s awgn function, which adds a white Gaussian noise of a certain power in dB, as specified by the user, to the measurements. The imaging algorithm properly reconstructs the signal when the the SNR is of the order of 20 dB; however, for SNR of 10 dB or below, the targets in the image cannot be distinguished. At an SNR of 15 dB, only the plastic cavity has yielded the correct results, whereas the images from the regular setup and the aluminum cavity include one or multiple strong artifacts. Therefore, it appears that the plastic cavity, in addition to improving the cross-range resolution, is the most adaptive case to noise.

## 5. Conclusions

In this work, a theoretical study of using solid cavities enclosing ultrasound sources to randomize the measurements was presented. Such a novel measurements scheme was combined with CS theory to retrieve the image of two objects using a reduced number of transmitters. It was shown that the sensing capacity and the PSF of the focused beam were significantly improved when a cavity, made of aluminum or plastic, was utilized. The recovered images of two point-like targets inside a uniform medium showed that the use of the cavity enhances the cross-range resolution, but they might still possess some weak artifacts when the SNR decays below 10 dB. The novelty of this work is the introduction of spectral coding cavities into ultrasound imaging. This work was limited by the simplified simulation layout that considered a small two-dimensional imaging domain, a reduced number of transceivers and a small number of targets to reduce the computational burden and to keep the assumptions valid. In future studies, a three-dimensional model, including the interactions between solids and acoustic waves, and a more complicated imaging domain with a heterogeneous background medium and larger targets will be considered. Furthermore, the effect of the sensor line array design or using a single sensor, as well as lower SNR values should be studied fully.

## Figures and Tables

**Table 1:** The acoustic properties of the materials used in the simulations.

Material	Density	Longitudinal Speed of Sound	Reference
Acrylic (PMMA)	1200 kg/m${}^{3}$	2730 m/s	[43,44]
Background	1035 kg/m${}^{3}$	1487 m/s	[45,46]
Target	1077 kg/m${}^{3}$	1549 m/s	[47,48]
Steel	7700 kg/m${}^{3}$	5050 m/s	[49]
Aluminum	2730 kg/m${}^{3}$	6800 m/s	[49]
VeroWhitePlus	1175 kg/m${}^{3}$	2539 m/s	[50,51]

**Table 2:** The geometric values used in the simulations.

Parameter	Value	Parameter	Value
$W_{D}$	6 mm	$h_{D}$	6 mm
$t_{cl}$	2 mm	$t_{im}$	2 mm
$t_{id}$	2 mm	$t_{c}$	0.05 mm
$d_{t}$	0.1 mm	$\left(x_{s,1},y_{s,1}\right)$	(−0.5, 2.9) mm
$\left(x_{s,2},y_{s,2}\right)$	(0.5, 2.9) mm	$\left(x_{t,1},y_{t,1}\right)$	(−0.6, −2) mm
$\left(x_{t,2},y_{t,2}\right)$	(0.8, −2) mm	$n_{x}$	501
$n_{y}$	120		

## Abbreviations

The following abbreviations are used in this manuscript:

CS	Compressive Sensing
SNR	Signal-to-Noise Ratio
ADMM	Alternating Direction Method of Multipliers
PSF	Point Spread Function

## Appendix A

Here, the method adopted to construct the sensing matrix in this work is presented. Equation (8) can be transformed into a matrix equation for a certain number of pixels *P* in the imaging domain and an available number of measurements *M*. If each pixel *p* has an infinitesimal small area $dA_{p}$ and the background fields $P_{b}^{T,i}$, originated from the *i*-th transmitter, are measured at the location of the *j*-th receiver, i.e., $r_{R,j}$, then (8) turns into:

(A1) $$P_{s}\left(r_{R,j},\omega \right)\approx \sum_{p=1}^{P}G_{b}\left(r_{R,j},r_{p},\omega \right)k_{b}^{2}\left(r_{p},\omega \right)P_{b}^{T,i}\left(r_{p},\omega \right)dA_{p}\;X\left(r_{p}\right),$$

in which $G_{b}\left(r_{R,j},r_{p},\omega \right)$ is Green’s function due to the *j*-th receiver, when it acts in transmission mode. The total number of transmitters and receivers is respectively denoted by $N_{T}$ and $N_{R}$ and thus $i\in \{1,\cdots ,N_{T}\}$ and $j\in \{1,\cdots ,N_{R}\}$. The number of frequencies by which the medium is insonified is $N_{f}$, and $q\in \{1,\cdots ,N_{f}\}$ is a counter for each frequency. Assuming noiseless simulations, the matrix form of the above equation (when all transmitters, receivers and frequencies at which the medium is insonified are considered) can be expressed as:

(A2) $$b_{\left(M\times 1\right)}=A_{\left(M\times P\right)}{\hat{x}}_{\left(P\times 1\right)},$$

where A is the sensing matrix, $\hat{x}$ is the unknown vector (the discretized version of X(r) at each pixel) and b is the measurement vector. In other words, $\hat{x}$ is the column-wise stacking of the values of the contrast parameter, in 2D space, at each pixel in the imaging domain. It will be shown below that $M=N_{RT}\times N_{f}$, where $N_{RT}=N_{R}\times N_{T}$. The sensing matrix for a homogeneous background can be constructed by separating the terms in (A1) that correspond to transmitters and receivers, individually, as is shown in the following:

(A3) $$A={\mathrm{diag}}_{M\times M}\left(dA_{p},\cdots ,dA_{p}\right)A_{T}\circ A_{R}$$

where:

(A4a) $$\begin{array}{cccc} A_{T} & =\left(\begin{array}{c} {\tilde{A}}_{T,1}\\ \vdots \\ {\tilde{A}}_{T,i}\\ \vdots \\ {\tilde{A}}_{T,N_{T}} \end{array}\right), & {\tilde{A}}_{T,i} & ={\left(\begin{array}{c} A_{T,i}\\ \vdots \\ A_{T,i}\\ \vdots \\ A_{T,i} \end{array}\right)}_{N_{R}\times 1}, \end{array}$$

(A4b) $$A_{T,i}=\left(\begin{array}{ccc} \begin{array}{c} k_{b}^{2}\left(r_{1},\omega_{1}\right)P_{b}^{T,i}\left(r_{1},\omega_{1}\right) \end{array} & \cdots & \begin{array}{c} k_{b}^{2}\left(r_{P},\omega_{1}\right)P_{b}^{T,i}\left(r_{P},\omega_{1}\right) \end{array}\\ \begin{array}{c} k_{b}^{2}\left(r_{1},\omega_{2}\right)P_{b}^{T,i}\left(r_{1},\omega_{2}\right) \end{array} & \cdots & \begin{array}{c} k_{b}^{2}\left(r_{P},\omega_{2}\right)P_{b}^{T,i}\left(r_{P},\omega_{2}\right) \end{array}\\ \vdots & \ddots & \vdots \\ \begin{array}{c} k_{b}^{2}\left(r_{1},\omega_{N_{f}}\right)P_{b}^{T,i}\left(r_{1},\omega_{N_{f}}\right) \end{array} & \cdots & \begin{array}{c} k_{b}^{2}\left(r_{P},\omega_{N_{f}}\right)P_{b}^{T,i}\left(r_{P},\omega_{N_{f}}\right) \end{array} \end{array}\right),$$

(A4c) $$\begin{array}{cccc} A_{R} & ={\left(\begin{array}{c} {\tilde{A}}_{R}\\ \vdots \\ {\tilde{A}}_{R}\\ \vdots \\ {\tilde{A}}_{R} \end{array}\right)}_{N_{T}\times 1}, & {\tilde{A}}_{R} & =\left(\begin{array}{c} A_{R,1}\\ \vdots \\ A_{R,j}\\ \vdots \\ A_{R,N_{R}} \end{array}\right), \end{array}$$

and

(A4d) $$A_{R,j}=\left(\begin{array}{ccc} \begin{array}{c} G_{b}\left(r_{R,j},r_{1},\omega_{1}\right) \end{array}\begin{array}{c} G_{b}\left(r_{R,j},r_{2},\omega_{1}\right) \end{array} & \cdots & \begin{array}{c} G_{b}\left(r_{R,j},r_{P},\omega_{1}\right) \end{array}\\ \begin{array}{c} G_{b}\left(r_{R,j},r_{1},\omega_{2}\right) \end{array}\begin{array}{c} G_{b}\left(r_{R,j},r_{2},\omega_{2}\right) \end{array} & \cdots & \begin{array}{c} G_{b}\left(r_{R,j},r_{P},\omega_{2}\right) \end{array}\\ \vdots & \ddots & \vdots \\ \begin{array}{c} G_{b}\left(r_{R,j},r_{1},\omega_{N_{f}}\right) \end{array}\begin{array}{c} G_{b}\left(r_{R,j},r_{2},\omega_{N_{f}}\right) \end{array} & \cdots & \begin{array}{c} G_{b}\left(r_{R,j},r_{P},\omega_{N_{f}}\right) \end{array} \end{array}\right),$$

with ${\mathrm{diag}}_{V\times V}\left(\alpha ,\cdots ,\alpha \right)$ denoting a diagonal matrix of size V×V, where the arbitrary parameter α is placed on the main diagonal and S∘Z denoting Hadamard or entry-wise multiplication of arbitrary matrices S and Z.

It can be noticed that each row of $A_{T,i}$ is formed by reshaping the numerically-calculated background pressure field times the wavenumber squared at each frequency $\omega_{q}$, generated by the *i*-th transmitter, and over a geometrical domain of size $n_{x}\times n_{y}$ into a row vector of size 1×P, where $P=n_{x}.n_{y}$. Similarly, each row of $A_{R,i}$ is generated by turning the numerically-computed Green’s function in the background over the same domain of size $n_{x}\times n_{y}$ into a row vector of size 1×P, when the receivers act as transmitters. In this study, only the exponential phase term of each element of the sensing matrix is used for imaging, similar to the method used in [56], and as shown, it generated the correct imaging results.
